# Recurrent Chronic Bronchitis

**Published:** 1875-04

**Authors:** 


RECURRENT CHRONIC BRONCHITIS.—Many persons are subject to attacks of chronic winter cough, commencing with difficult breathing and a hard dry cough. In this stage, says Dr. Lawrie, of Glasgow, no remedy gives such prompt relief as *iodide of potassium.* It promotes and restores the bronchial secretion, and tranquilizes the respiration. When free secretion has set in, it should be abandoned.

